# Comparative microRNA signatures based on liquid biopsy to identify lymph node metastasis in T1 colorectal cancer patients undergoing upfront surgery or endoscopic resection

**DOI:** 10.1038/s41420-025-02348-5

**Published:** 2025-02-20

**Authors:** Kazuaki Okamoto, Hiroaki Nozawa, Tsuyoshi Ozawa, Yoko Yamamoto, Yuichiro Yokoyama, Shigenobu Emoto, Koji Murono, Kazuhito Sasaki, Mitsuhiro Fujishiro, Soichiro Ishihara

**Affiliations:** 1 Department of Surgical Oncology, The University of Tokyo, Tokyo, Japan; 2 Department of Translational Molecular Medicine, Division of Molecular Oncology, Saint John’s Cancer Institute at Providence Saint John’s Health Center, Santa Monica, CA USA; 3 Department of Gastroenterology, The University of Tokyo, Tokyo, Japan

**Keywords:** Colon cancer, Rectal cancer

## Abstract

After endoscopic resection of T1 colorectal cancer (CRC) with a high risk of lymph node metastasis (LNM), additional surgery is required. However, the actual frequency of LNM based on conventional risk factors is less than 16%. There is a need for biomarkers to identify T1 CRC carrying a high risk of metastasis to avoid unnecessary radical surgery. Based on the comparison of serum miRNA between stage I/II and stage III from a large-scale in silico dataset, we conducted a validation analysis of the selected miRNAs using plasma samples from LNM-positive and LNM-negative T1 CRC patients who underwent endoscopic treatment followed by radical surgery at our hospital. In the validation cohort, the three-miRNA classifiers (miR-195-5p, miR-221-3p, and miR-193b-3p) effectively identified LNM-positive T1 CRC patients who received upfront surgery with an area under the curve (AUC) value of 0.74. Moreover, in T1 CRC patients after endoscopic resection, miR-195-5p and miR-221-3p were able to predict LNM with an AUC of 0.74. Plasma miRNA signatures may serve as effective predictors for LNM in T1 CRC both before upfront surgery and after endoscopic resection.

## Introduction

Colorectal cancer (CRC) is a leading cause of cancer-related mortality worldwide [1, 2]. Early detection and accurate staging are crucial for optimal management and improved patient outcomes. In recent years, there has been a notable increase in the diagnosis of T1 CRCs, which are invasive submucosal lesions, owing to the widespread implementation of CRC screening programs and advancements in endoscopic imaging technologies [3–5]. This surge has prompted a paradigm shift in the clinical approach to T1 CRCs [6, 7].

Traditionally, patients diagnosed with T1 CRCs were treated with radical surgical resection. However, the advent of sophisticated endoscopic techniques, such as endoscopic submucosal dissection and endoscopic mucosal resection, has revolutionized the management landscape [8]. These minimally invasive procedures now offer curative treatment options for a significant proportion of T1 CRC patients, circumventing the need for extensive surgical interventions and their associated morbidities.

Nonetheless, the decision to pursue endoscopic or surgical treatment depends on a critical factor: the accurate assessment of lymph node metastasis (LNM) risk. Current pathological criteria employed to identify high-risk patients include depth of submucosal invasion exceeding 1000 μm, presence of lymphovascular invasion, poorly differentiated histology, and budding grade [9–13]. When at least one of these factors is present, patients are deemed high-risk and recommended for radical surgery [9]. Paradoxically, while this approach classifies approximately up to 80% of T1 CRC patients as high-risk, postoperative pathological analysis reveals that only 6–16% of these individuals truly have LNM [14–18].

This striking discordance between preoperative risk stratification and actual metastatic involvement underscores the pressing need for more reliable and accurate biomarkers. Existing pathological criteria lack the precision to discriminate between genuinely high-risk patients requiring surgical intervention and those who could be spared from unnecessary, morbid procedures. The development of robust molecular signatures capable of reliably identifying LNM preoperatively is paramount to optimizing clinical decision-making and reducing the considerable burden of overtreatment in this patient population.

Accumulating evidence suggests that microRNAs (miRNAs), small non-coding RNA molecules that regulate gene expression, hold significant promise as cancer biomarkers [19–22]. Several studies have demonstrated the differential expression of specific miRNAs in CRC pathogenesis, emphasizing their potential as non-invasive diagnostic and prognostic indicators [22–24]. However, their utility in stratifying LNM risk in T1 CRC remains controversial. In addition, although there were several attempts to predict LNM in CRC using miRNA from resected specimens or sera collected before surgery regardless of endoscopic treatments [25–29], there have been no reports on blood-based miRNA that can predict LNM after endoscopic removal of the primary tumor. There is an urgent need for tools that can predict LNM in CRC even in the absence of a primary tumor following endoscopic resection. Therefore, the present study aimed to explore miRNAs in a minimally invasive, circulating “liquid biopsy” assay, thereby providing an accurate, non-invasive tool for preoperative risk stratification in T1 CRC patients who initially received endoscopic resection as well as those who underwent upfront surgery.

## Results

### Selection of miRNAs for LNM prediction based on serum-based in silico data

**Fig. 1:**
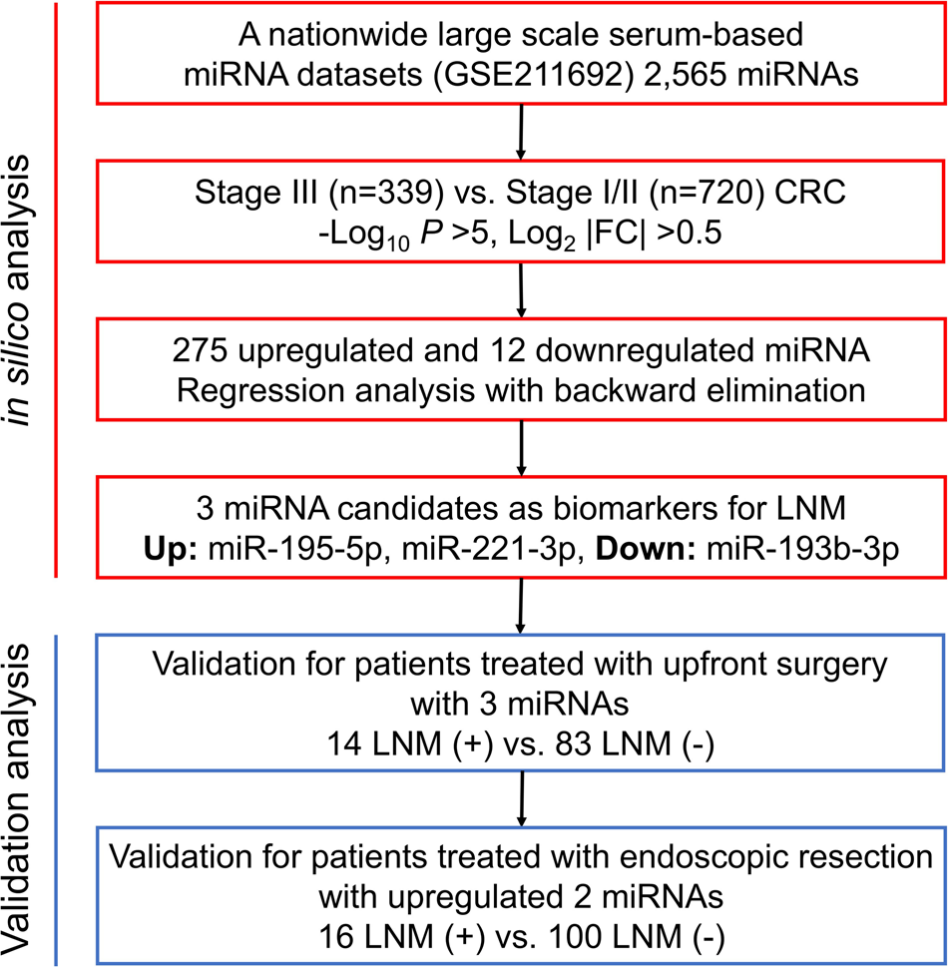
Study flow diagram for analyses.

**Fig. 2:**
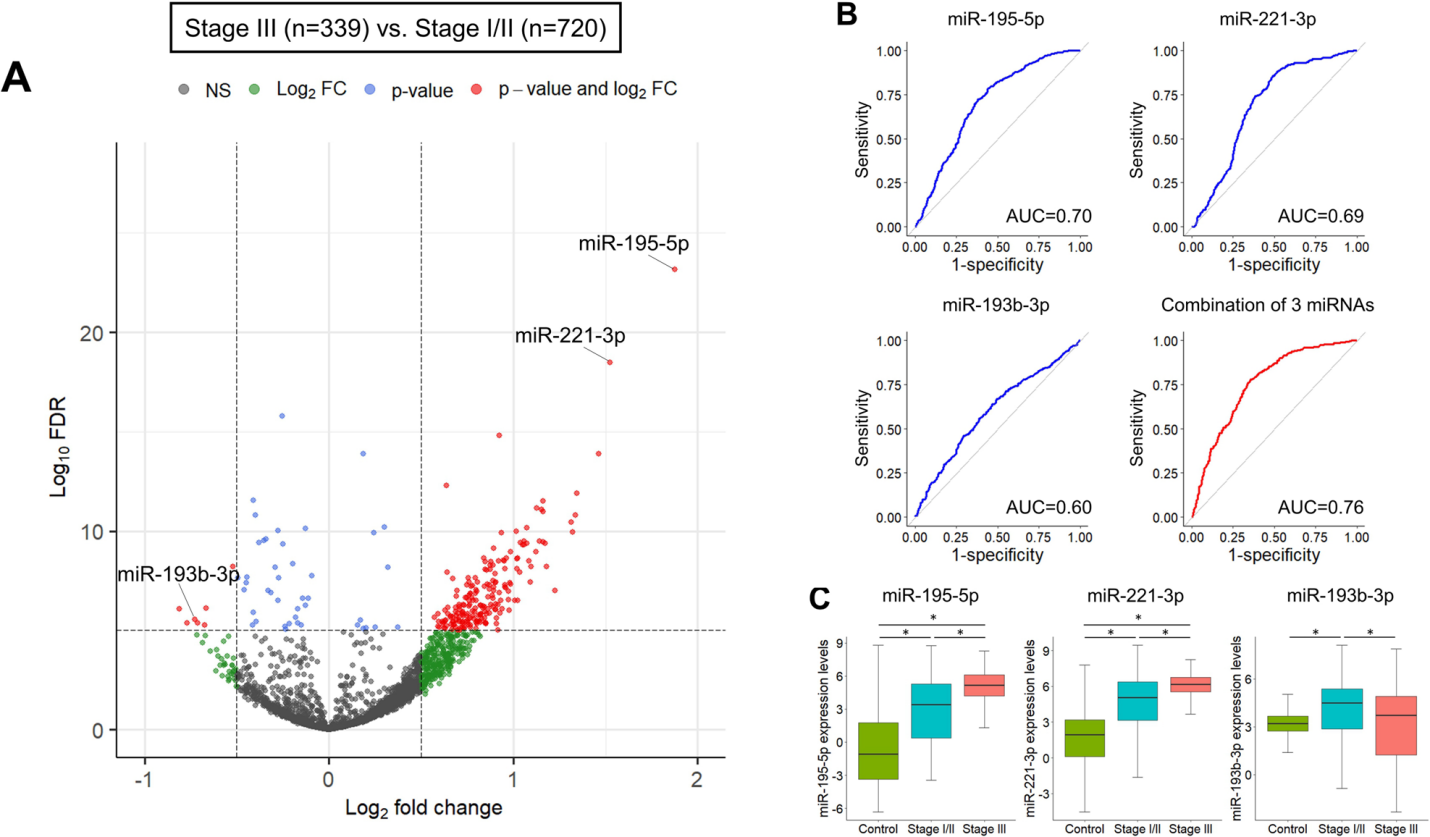
in silico analysis of GSE211692. **A** Volcano plot of serum miRNAs comparing stage III and stage I/II CRC patients. **B** ROC curve analysis to evaluate the predictive ability for LNM of three individual miRNAs and the combination panel. **C** Expression levels of three miRNAs in the control group, stage I/II CRC patients, and stage III CRC patients.

The flow of this study is shown in Fig. 1. First, we compared the serum miRNAs of 339 stage III CRC patients and 720 stage I/II CRC patients using the GSE211692 dataset. The clinical backgrounds of the patients are presented in Supplementary Table 1. Out of 2565 miRNAs, 275 were upregulated and 12 were downregulated in stage III CRC (Fig. 2A). To identify a miRNA signature specific to LNM, we selected the top 5 upregulated and downregulated miRNAs, and variable selection through regression analysis identified two upregulated miRNAs (miR-195-5p and miR-221-3p) and one downregulated miRNA (miR-193b-3p) (Supplementary Table 2). The combination of these three miRNAs allowed for the discrimination of stage III CRC patients with an area under the curve (AUC) of 0.76 in the ROC analysis (Fig. 2B).

Compared to non-cancer patients, miR-195-5p and miR-221-3p showed a progressive increase with advancing stages, whereas miR-193b-3p did not show a significant difference between the control group and stage III CRC patients (Fig. 2C). Assuming that patients without LNM after endoscopic resection would exhibit a miRNA signature similar to non-cancer patients, we evaluated the predictive ability of the three miRNAs for LNM in the upfront surgery group and the two upregulated miRNAs in the endoscopic resection group.

### Prediction of LNM in patients undergoing upfront surgery

**Fig. 3:**
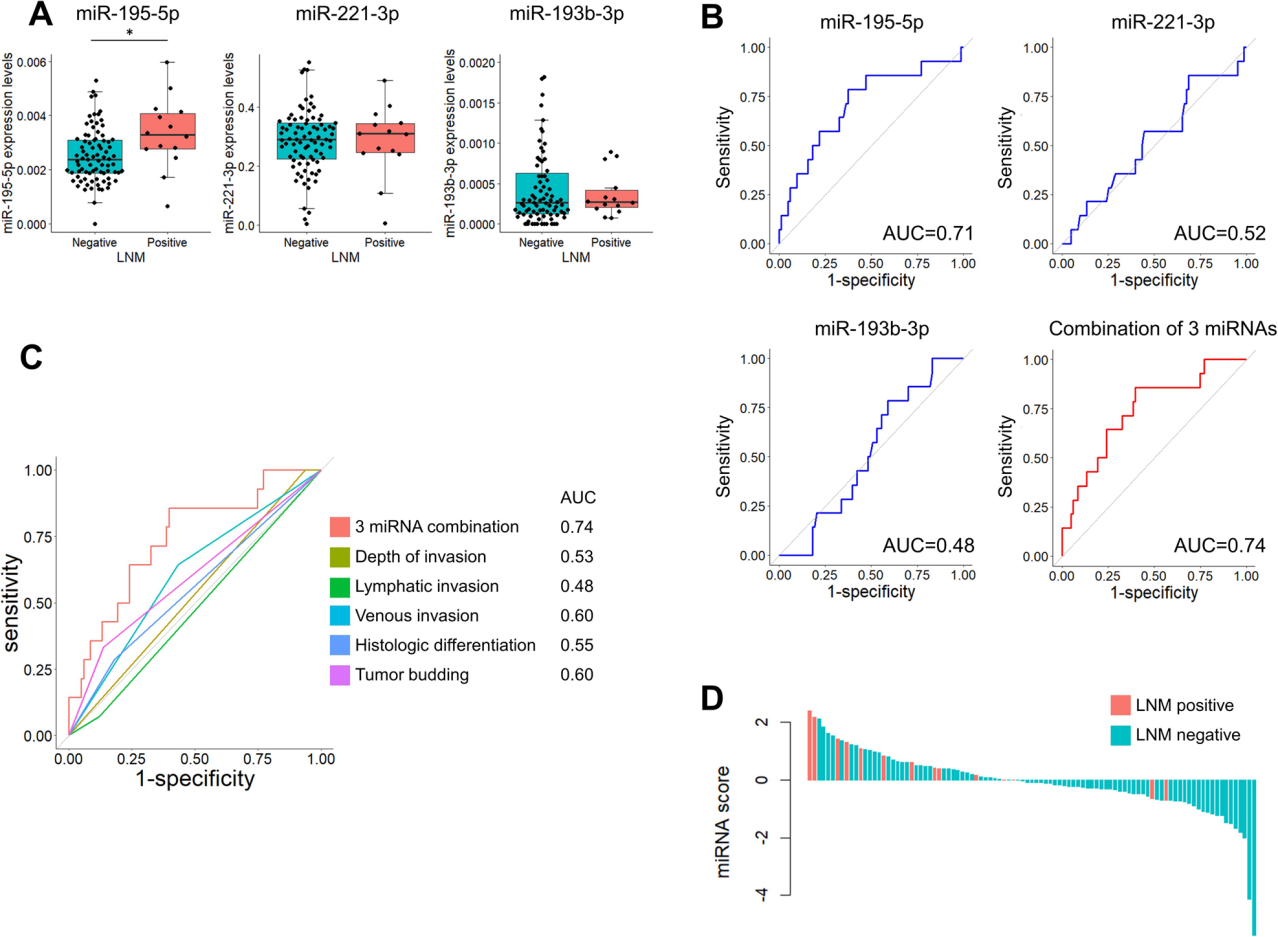
Analysis of CRC patients treated with upfront surgery. **A** Expression levels of three miRNAs according to LNM. **B** ROC curve analysis to evaluate the predictive ability for LNM of three individual miRNAs and the combination panel. **C** ROC curve analysis comparing the predictive ability for LNM between the miRNA signature panel and conventional risk factors. **D** Waterfall plot for miRNA score distribution.

Among 97 patients who underwent upfront surgery and were pathologically diagnosed with T1 cancer, 14 were found to have LNM. No differences were observed in the clinicopathological factors of patients based on the presence or absence of LNM (Table 1). Among the three miRNAs, miR-195-5p showed a significant increase in LNM-positive patients (*p* = 0.012), whereas miR-221-3p and miR-193b-3p did not exhibit significant changes based on the presence of LNM (*p* = 0.82 and *p* = 0.79, respectively; Fig. 3A). Therefore, miR-195-5p achieved a diagnostic accuracy for LNM with an AUC of 0.71. In contrast, miR-221-3p had an AUC of 0.52, and miR-193b-3p had an AUC of 0.48. The following miRNA score was calculated based on the expression levels of each miRNA:

$${\rm{miRNA\; score\; for\; upfront\; surgery}}{\rm{group}}=({\rm{miR}}-195-5{\rm{p}})* 817+({\rm{miR}}-193{\rm{b}}-3{\rm{p}})* (-981)+({\rm{miR}}-221-3{\rm{p}})* (-2.42)-0.999$$

**Table 1:** Clinicopathological parameters of patients in the upfront surgery group according to the presence of LNM.

Variable	LNM positive (*n* = 14)	LNM negative (*n* = 83)	*p*-value
Demographic data			
Age, years	62 (49–76)	66 (37–94)	0.64
Sex, male	7 (50%)	43 (52%)	0.90
Tumor location			0.35
Cecum	3 (21%)	13 (16%)	
Ascending colon	1 (7%)	20 (24%)	
Transverse colon	0 (0%)	5 (6%)	
Descending colon	0 (0%)	6 (7%)	
Sigmoid colon	5 (36%)	13 (16%)	
Rectum	5 (36%)	26 (31%)	
Tumor size, mm	21 (8–35)	19 (5–53)	1.0
Tumor type			0.71
Polypoid	6 (43%)	41 (49%)	
Flat	1 (7%)	11 (13%)	
Depressed	7 (50%)	31 (37%)	
Tumor histology			0.41
Well	5 (36%)	45 (54%)	
Moderate	5 (36%)	23 (28%)	
Poor	4 (29%)	15 (18%)	
Tumor depth (µm)			0.11
<1000	0 (0%)	5 (6%)	
1000–1999	0 (0%)	58 (70%)	
≥2000	13 (93%)	17 (20%)	
Unavailable	1 (7%)	3 (4%)	
Lymphatic invasion			1.0
Positive	1 (7%)	10 (12%)	
Negative	13 (93%)	73 (88%)	
Vascular invasion			0.15
Positive	9 (64%)	36 (43%)	
Negative	5 (36%)	47 (57%)	
Tumor budding			0.13
Grade 1	8 (57%)	63 (76%)	
Grade 2	2 (14%)	8 (10%)	
Grade 3	2 (14%)	2 (2%)	
Unavailable	2 (14%)	10 (12%)	

Values are presented as the number of patients (%) or the median (range).

While miR-221-3p and miR-193b-3p were not effective individually, their combination with miR-195-5p increased the discriminative accuracy to an AUC of 0.74 (95% confidence interval [CI]: 0.60–0.88) (Fig. 3B), which is relatively high although not statistically significant compared to conventional risk factors for LNM. According to the miRNA score, among 45 patients classified as LNM positive, 12 were truly LNM positive (27%; Fig. 3D); in contrast, the true-positive rate was 14% by the conventional criteria. The miRNA signature identifier demonstrated a sensitivity of 0.86, a specificity of 0.60, and an overall diagnostic accuracy of 0.64 (Supplementary Table 3).

### Prediction of LNM in patients undergoing endoscopic resection

**Fig. 4:**
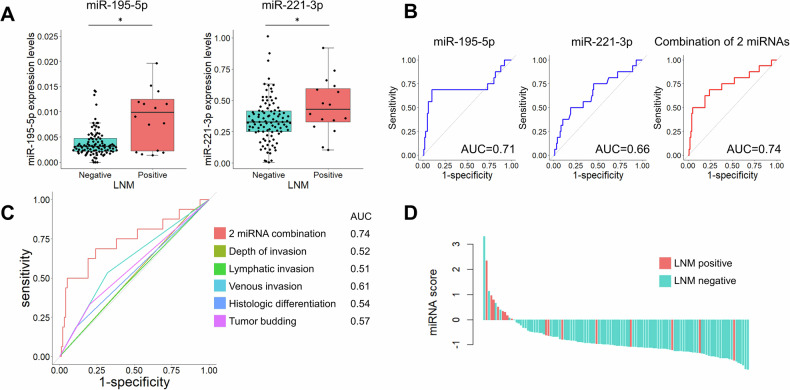
Analysis of CRC patients treated with prior endoscopic resection. **A** Expression levels of three miRNAs according to LNM. **B** ROC curve analysis to evaluate the predictive ability for LNM of three individual miRNAs and the combination panel. **C** ROC curve analysis comparing the predictive ability for LNM between the miRNA signature panel and conventional risk factors. **D** Waterfall plot for miRNA score distribution.

Next, we proceeded with the analysis of patients who underwent endoscopic resection. Among 116 patients diagnosed with T1 cancer who met conventional risk factors, additional surgical resection was performed, resulting in 16 patients diagnosed with LNM. Regarding clinical and pathological factors of these patients, LNM-positive patients showed a higher frequency of vascular invasion (50% vs. 32%, *p* = 0.027) and were more likely to have high-grade budding (*p* = 0.004), as shown in Table 2. Both miR-195-5p and miR-221-3p showed increased levels in LNM-positive patients (*p* = 0.008 and *p* = 0.045, respectively; Fig. 4A), with predictive abilities of AUC = 0.71 for miR-195-5p and AUC = 0.66 for miR-221-3p. The following miRNA score was calculated based on the expression levels of the individual miRNAs:

$${\rm{miRNA\; score\; for\; prior\; endoscopic\; resection\; group}}=({\rm{miR}}-195-5{\rm{p}})* 91.7+({\rm{miR}}-221-3{\rm{p}})* 2.1-2.06$$

**Table 2:** Clinicopathological parameters of patients in prior endoscopic resection group according to the presence of LNM.

Variable	LNM positive (*n* = 16)	LNM negative (*n* = 100)	*p*-value
Demographic data			
Age, years	64 (51–83)	67 (39–87)	0.95
Sex, male	9 (56%)	52 (52%)	0.75
Tumor location			0.97
Cecum	0 (0%)	3 (3%)	
Ascending colon	3 (19%)	16 (16%)	
Transverse colon	2 (13%)	12 (12%)	
Descending colon	0 (0%)	2 (2%)	
Sigmoid colon	5 (31%)	25 (25%)	
Rectum	6 (38%)	42 (42%)	
Tumor size, mm	15 (6–45)	13 (3–45)	0.55
Tumor type			0.62
Polypoid	11 (69%)	65 (65%)	
Flat	4 (25%)	19 (19%)	
Depressed	1 (6%)	16 (16%)	
Tumor histology			0.48
Well	6 (38%)	37 (37%)	
Moderate	3 (19%)	12 (12%)	
Poor	7 (44%)	51 (51%)	
Tumor depth (µm)			0.37
<1000	1 (6%)	11 (11%)	
1000–1999	3 (19%)	21 (21%)	
≥2000	9 (56%)	62 (62%)	
Unavailable	3 (19%)	6 (6%)	
Lymphatic invasion			0.90
Positive	7 (44%)	42 (42%)	
Negative	9 (56%)	58 (58%)	
Vascular invasion			0.027
Positive	8 (50%)	32 (32%)	
Negative	7 (44%)	68 (68%)	
Unavailable	1 (6%)	0 (0%)	
Tumor budding			0.004
Grade 1	8 (50%)	72 (72%)	
Grade 2	0 (0%)	14 (14%)	
Grade 3	4 (25%)	4 (4%)	
Unavailable	4 (25%)	10 (10%)	

Values are presented as the number of patients (%) or the median (range).

When the two miRNAs were combined, they achieved an AUC of 0.74 (95% CI: 0.58–0.89; Fig. 4B). Comparison with conventional risk factors showed similar trends as observed in the upfront surgery group (Fig. 4C). Among 13 patients classified as LNM positive with the miRNA score, 8 were truly LNM positive (62%); in contrast, the true-positive rate was 14% by the conventional criteria. The miRNA signature classifier for the prior endoscopic resection group exhibited a sensitivity of 0.50, a specificity of 0.95, and an overall diagnostic accuracy of 0.89 (Supplementary Table 3).

## Discussion

Various studies have been conducted on the evaluation of LNM after endoscopic treatment for T1 CRC [13–18], and recent reports have highlighted the use of miRNA for diagnosis [25–29]; Jung et al. and Ozawa et al. reported that LNM could be predicted from miRNAs in tumor tissues of T1 cancer [25, 26]. Subsequently, Qu et al. demonstrated that LNM in CRC, regardless of depth, could be predicted with an AUC of 0.764 using a model based on four serum miRNAs (miR-122-5p, miR-146-5p, miR-186-5p, and miR-193a-5p) [27]. Following this, Wada et al. developed a model incorporating four serum miRNAs (miR-181b, miR-193b, miR-195, and miR-411) and five mRNAs achieving an AUC of 0.82 for predicting LNM in high-risk T1 CRC [28]. Moreover, Miyazaki et al. from the same team reported that analyzing miRNAs in serum exosomes could predict LNM with high accuracy (AUC = 0.84) [29]. However, these reports on blood-based miRNAs have only evaluated the serum of cancer-bearing patients who underwent upfront surgery. It remains unclear whether the same miRNA signatures are applicable to patients with small lesions amenable to endoscopic resection or to patients after endoscopic resection.

Previous studies predicting LNM in T1 CRC with serum miRNA have developed miRNA signatures by utilizing miRNAs identified in cancer tissue [26, 28, 29]. In contrast, our study constructed a miRNA signature based on a large-scale blood miRNA database, which may allow for the evaluation of miRNAs that are truly altered due to LNM. The signature for predicting LNM comprised only a few miRNAs in the current study; particularly, only two miRNAs were used for the group that underwent endoscopic resection. This makes it relatively inexpensive to measure and highly versatile. Moreover, our study stands out in simultaneously evaluating patients who underwent upfront surgery and those who underwent endoscopic resection. Although validation with an independent cohort has not been performed, we have examined more than 200 cases in this study, which is one of the largest sample sizes compared to previous reports on miRNA-based LNM diagnosis.

We have identified miR-195-5p as the most promising predictor for LNM, which is effective both in cancer-bearing states and post-endoscopic resection. This conclusion is supported by the large sample size of in silico data and previous reports indicating elevated miR-195-5p levels in tumor tissues or blood of LNM-positive CRC patients [26, 28, 29]. miR-195-5p has been reported as a tumor-suppressive miRNA in CRC and other cancer types [30]. miR-195-5p is shown to regulate pro-tumorigenic signaling pathways such as the Hippo pathway and NOTCH pathway [30–32]. Consequently, low expression of miR-195-5p in tumor tissues has been associated with poor outcomes [30, 33, 34]. In this study, we observed an increase in the expression of miR-195-5p in the plasma of patients who were LNM positive. This contradicts previous reports that miR-195-5p tends to decrease in advanced stages, including LNM-positive patients [34]. However, it’s possible that the increase in miR-195-5p observed in our study could be a reactive response associated with tumor growth and metastasis.

miR-221-3p is known to play a pro-tumorigenic role in various cancer types [35–37], and in CRC, it has been reported that tumor-derived miR-221-3p contributes to processes such as angiogenesis [38]. While miR-221-3p did not serve as an indicator for distinguishing LNM before upfront surgery, it proved effective after endoscopic resection. This suggests that miR-221-3p is a sensitive marker whose expression increases when tumor remnants, even microscopic ones, persist in lymph nodes compared to a tumor-free state. The lack of significant difference in the upfront surgery group could be due to our study model; we compared stage I/II with stage III in the in silico analysis without considering the specific T stages within these stage groups. The advanced T stages in the stage III group, besides the N stage, might have influenced the expression of miR-221-3p.

miR-193b-3p has been reported to function as an oncomiR contributing to tumor progression in CRC [39]. Our in silico analysis revealed that while miR-193b-3p expression increases in stage I/II patients, it decreases as the disease progresses. On the other hand, in our study’s upfront surgery group, miR-193b-3p alone did not effectively identify LNM, suggesting that, like miR-221-3p, its expression may also depend on the tumor’s T stage. However, combining miR-193b-3p with miR-195-5p improved the accuracy of LNM detection, similar to the findings from our in silico analysis. Therefore, incorporating miR-193b-3p into a miRNA panel to support miR-195-5p could be beneficial. Notably, previous studies reported increased miR-193b-3p expression in tumors and serum of LNM-positive T1 CRC patients [26, 28, 29]. This discrepancy might be due to the influence of non-malignant conditions such as diabetes or liver diseases on blood miR-193b-3p levels, as these conditions are known to affect its expression [40, 41]. Further research is needed to determine the effectiveness of miR-193b-3p in identifying LNM in CRC.

In this study, we established the cutoff for the miRNA score to maximize the AUC value of the ROC. Although the diagnostic accuracy of the miRNA signature identifier in the prior endoscopic resection group was notably high at 0.89, the sensitivity was 0.50. For appropriate additional surgery, a high sensitivity is ideal to ensure that patients with lymph node metastasis are not overlooked. Therefore, it may be necessary to evaluate more cases to determine an appropriate cutoff value.

This study has several limitations. Firstly, it is a single-institution study, and we have not been able to validate the miRNA signature score developed from our cohort; there is a possibility of overfitting. Secondly, the in silico analysis used in this study is serum-based, whereas our cohort is plasma-based; changes in miRNA due to the coagulation process in serum may result in a miRNA signature that differs from that in plasma. Additionally, although we selected miRNAs with a focus on versatility, we might have eliminated miRNAs that were originally useful. Lastly, our miRNA-based LNM prediction model may include patients who are truly positive for LNM but were classified as negative by the model. Therefore, the cutoff values for the miRNA score should be set carefully, and treatment decisions should consider other risk factors as well.

In conclusion, our discovered miRNA combination panel effectively predicts LNM in T1 CRC patients, both prior to upfront surgery and following endoscopic resection, with relatively good accuracy. Integrating miRNA measurements alongside existing risk factors enhances the precision of LNM prediction, suggesting the potential to avoid unnecessary surgeries in patients after endoscopic resection.

## Materials and methods

### Candidate miRNA selection and miRNA signature construction

We analyzed and developed miRNA signatures using a large, publicly available serum miRNA dataset GSE211692 from the Gene Expression Omnibus, which includes various types of cancers, including CRC, as well as non-cancer individuals [42]. To identify a miRNA signature specific to LNM, we compared serum miRNA profiles between stage I/II CRC patients (without LNM) and stage III patients (with LNM). miRNAs upregulated or downregulated in stage III CRC samples were evaluated as candidate biomarkers. miRNAs that met the criteria of −log_10_(FDR) > 5 and log_2_|fold change | > 0.5 were considered to have significant differences. To avoid overfitting in our cohort validation, we rigorously selected the obtained miRNAs using backward elimination methods [43]. Additionally, we compared the values of each miRNA between non-cancer individuals and CRC patients. Subsequently, the detective power of this miRNA panel was evaluated in clinical cohorts.

### Patients and sample collection

We investigated patients who underwent surgical treatment for T1 CRC at our hospital between January 2015 and October 2019. The study included patients diagnosed with pathological T1 cancer following curative resection, as well as those with T1 cancer who underwent additional resection due to LNM risk after prior endoscopic resection. We excluded cases with inappropriate sample handling before RNA extraction, patients with concurrent other cancers, individuals with familial adenomatous polyposis, and those with concomitant inflammatory bowel disease. We retrieved the following data: sex, age, the primary tumor location, and pathological factors such as tumor size, morphology, histology, depth of invasion, lymphatic invasion, vessel invasion, and budding grade. The TNM pathological classification of tumors at diagnosis was determined according to the American Joint Committee on Cancer staging manual [44]. The present study was approved by the Ethics Committees of the University of Tokyo (3252 and G3552). Informed consent for sample collection, analysis, and publication was obtained from all patients involved in the study.

### Extraction and quantification of miRNAs

The patients’ blood samples were collected at the following time points: (i) for the group that underwent endoscopic resection, blood was drawn immediately before additional surgical resection, and (ii) for the group that did not undergo endoscopic resection, blood was drawn immediately before primary resection. The blood samples were collected using EDTA tubes, and plasma was extracted after centrifugation and stored at −80 °C. Plasma samples were filtered through a Minisart 0.8 μm filter (Sartorius, Göttingen, Germany), followed by miRNA extraction from 200 µL of plasma using the miRNeasy Serum/Plasma Advanced Kit (Qiagen, Venlo, The Netherlands) according to the manufacturer’s instructions. Subsequently, reverse transcription was conducted using the miRCURY LNA RT Kit (Qiagen). Quantification of miRNA was performed using the miRCURY LNA SYBR Green PCR Kit (Qiagen) with miRCURY LNA miRNA PCR Assays specific to each miRNA on the Applied Biosystems 7500 Fast Real-Time PCR System (Thermo Fisher Scientific, MA). Data analysis was carried out using the 7500 Software version 2.3 (Thermo Fisher Scientific), and miR-16-5p was used as an internal control for normalization [20].

### Verification of the ability of miRNA to predict LNM

Based on the analysis of in silico data, we validated the predictive ability of the candidate miRNAs for LNM in two groups: the upfront surgery group and the prior endoscopic resection group. The Receiver Operating Characteristic (ROC) analysis was used to evaluate the ability of the miRNA combination to distinguish LNM in both groups. For each group in our cohort, we applied a logistic regression model with each miRNA as a variable and set the resulting probability calculation formula as the miRNA score accordingly. The cutoff value to separate positive and negative cases was set to maximize the sum of sensitivity and (1 - specificity). Additionally, we compared the predictive ability of these miRNAs with conventional LNM risk factors, including tumor depth of invasion, vascular invasion, histological differentiation, and budding grade.

### Statistical analysis

Statistical analyses were performed using JMP Pro 17.3.0 (SAS Institute, Cary, NC) and R software (version 4.4.0; https://www.r-project.org/; R Foundation for Statistical Computing, Vienna, Austria). All variables were summarized as medians (range), means ± standard deviations, or numbers (percentages). Quantitative variables were compared using the Mann–Whitney *U*-test. Qualitative variables were compared using the chi-squared test with Yates’ correction. All reported *p*-values were two-sided and considered significant if less than 0.05.

## Supplementary information

Supplementary table

## Data Availability

The datasets during and/or analyzed during the current study are available from the corresponding author upon reasonable request. The in silico data analyzed in this study were obtained from Gene Expression Omnibus (GEO) at GSE211692.

## Patient and public involvement

We conducted a series of workshops with the public to discuss the potential impact of our findings and to gather opinions on the dissemination of results.
