# Supplementary material for: Comparative microRNA signatures based on liquid biopsy to identify lymph node metastasis in T1 colorectal cancer patients undergoing upfront surgery or endoscopic resection
Source: Cell Death Discov. 2025 Feb 20;11:67. doi: 10.1038/s41420-025-02348-5 (PMC11840149; doi:10.1038/s41420-025-02348-5)
Supplement: Supplementary file 1 — Supplementary table [file 41420_2025_2348_MOESM1_ESM.docx]

Supplementary Table 1

Demographic data of patients in GSE211692.

| Variable | Control (n = 5643) | Stage I/II (n = 720) | Stage III (n = 339) | *p*-value |
| --- | --- | --- | --- | --- |
| Age, years | 72 (20-100) | 65 (27-94) | 64 (24-87) | <0.001 |
| Sex, male | 2468 (44%) | 422 (59%) | 194 (57%) | <0.001 |

Values are presented as the number of patients (%) or the median (range).

Supplementary Table 2

miRNA candidates that distinguish stage III from stage I/II in GSE211692.

| miRNA | Status | Gap of expression (log_2_FC) | *p*-value |
| --- | --- | --- | --- |
| miR-195-5p | Upregulated | 1.88 | 2.6*10^-27^ |
| miR-221-3p | Upregulated | 1.52 | 2.7*10^-22^ |
| miR-193b-3p | Downregulated | -0.73 | 2.2*10^-7^ |

Values are presented as the number of patients (%) or the median (range).

FC: fold change.

Supplementary Table 3

Diagnostic accuracy of miRNA score models.

| Group | Upfront surgery (n = 97) | Prior endoscopic resection (n = 116) |
| --- | --- | --- |
| Sensitivity | 0.86 | 0.50 |
| Specificity | 0.60 | 0.95 |
| False positive rate | 0.40 | 0.05 |
| False negative rate | 0.14 | 0.50 |
| Accuracy | 0.64 | 0.89 |
